# Traditional and Unconventional Dried Fruit Snacks as a Source of Health-Promoting Compounds

**DOI:** 10.3390/antiox8090396

**Published:** 2019-09-13

**Authors:** Dario Donno, Maria Gabriella Mellano, Isidoro Riondato, Marta De Biaggi, Harilala Andriamaniraka, Giovanni Gamba, Gabriele Loris Beccaro

**Affiliations:** 1 Dipartimento di Scienze Agrarie, Forestali e Alimentari, Università degli Studi di Torino, 10095 Grugliasco (TO), Italy; gabriella.mellano@unito.it (M.G.M.); isidoro.riondato@unito.it (I.R.); marta.debiaggi@unito.it (M.D.B.); giovanni.gamba@unito.it (G.G.); gabriele.beccaro@unito.it (G.L.B.); 2 Mention Agriculture Tropicale et Développement Durable - Ecole Supérieure des Sciences Agronomiques, Université d’Antananarivo, 101 Antananarivo, Madagascar; jharilala@gmail.com

**Keywords:** *Malus domestica*, *Actinidia deliciosa*, *Diospyros kaki*, *Lycium barbarum*, phytochemicals, HPLC, dried fruit products

## Abstract

Dried fruits are important, healthy and popular snacks, despite the limited information on their nutritional profiles and phytochemical composition. The present work was aimed to study the chemical composition of freeze-dried fruits from four fruit species: two common commercial snacks (apple and goji) and two innovative products (kaki and kiwi). Sugar and organic acid levels, total phenolics (TPC), and main health-promoting phytochemicals were studied by HPLC fingerprinting analysis. Furthermore, *in vitro* antioxidant capacity (AOC) was preliminarily observed in these products. A Principal Component Analysis (PCA) was carried out as a multivariate approach as well. The TPC ranged from 210.9 mg GAE/100g DW (kiwi) to 872.6 mg GAE/100g DW (kaki), while dried fruit antioxidant capacity ranged from 23.09 mmol Fe^2+^/kg DW (goji) to 137.5 mmol Fe^2+^/kg DW (kaki). The most important phytochemical class in apple (two cultivars), kiwi, and kaki dried fruits was phenolics (from 74.6% to 93.3%), while monoterpenes were the first class in goji (67.5%). No anthocyanins have been identified in dried fruits because these compounds are most likely converted to phenolic acids during the drying process. This research intended to stimulate large-scale exploitation of commercial dried fruits as functional foods as well.

## 1. Introduction

Fruits confer basic nutrition and significant health benefits to humans, but many common fruits are seasonally produced and therefore they may not be available to consumers in fresh conditions during all the year. Thus, fresh fruits are processed or dried to extend their shelf life. In dried fruits, most of water content has been removed by several drying techniques and molecules are concentrated when compared to their quantity in fresh fruits [1]. Dehydration is one of the best methods for agricultural product preservation and for extending fruit shelf life [2]. The method is based on a complex process involving simultaneous heat and mass transfer [3]. The traditional drying method is based on solar energy, but environmental conditions may influence the process and cause a high loss in fruit quality [4]. Moreover, high temperatures are used by the main conventional techniques (traditional stoves) during fruit processing; for this reason, lower temperatures (freeze-drying) and/or decreased drying times are used in new techniques. The freeze-dried fruits are very crispy and light and they retain original flavour (e.g., aroma and taste) and bioactive molecules [5].

Grapes, berries, apricots, and plums are often dried whole, while mangoes, papayas, apples, and kiwifruits are processed as slices. Other fruits are dried in halves. Dried fruits can be easily stored and distributed during all the year, and they can be considered as healthier products compared to traditional snacks (both salty and sugary) worldwide [6]. Local food stores and markets offer many traditional dried fruit products (e.g., apples, figs, dates, goji, and apricots) together with innovative snacks as kiwifruits, kaki, berries, and papayas.

Dried fruits are rich in essential health-promoting substances and nutrients and their consumption is closely correlated with diet quality. These snacks may exert several health effects on humans, thanks to the synergistic combinations of their nutritional and bioactive compounds (“phytocomplex”)—in particular against obesity, type II diabetes, osteoporosis, cardiovascular diseases, and cancer [7]. In particular, dried fruit products are essential sources of dietary fibre and potassium with low levels of fat (0.32–0.93 g per 100 g) [1]. They are rich in sugars such as fructose and glucose, and carbohydrates, with low (<55) or moderate (from 56 to 69) insulin and glycaemic indices, comparable to fresh fruit ones, as demonstrated by different studies [8,9]. Low levels of insulin/glycaemic index are due to the presence of several substances (e.g., phenolic acids, tannins, and fibre) which may moderate the glycaemic and insulin response [5]. Both specific methods (e.g., high performance liquid chromatography coupled to different detection systems) and non-specific method (e.g., anthocyanins and total polyphenols by colorimetric assays) have been utilised for the characterisation of different molecules in dried fruits. In any case, literature on the phytochemical profiles of these products are still scarce. Moreover, the identified compounds and their related antioxidant capacity varies considerably according to genotype (e.g., cultivars) as well as pedoclimatic conditions and potentially used agrotechniques in plant material and derived-products as dried fruit snack [10,11].

Few data are available on the potential industrial uses of dried fruit snacks and their by-products. They can be used as an ingredient in bakery and confectionery products or they can be added or mixed to several cereal-based products, yogurts, and cheeses [12]. Moreover, further research could be performed in order to study the potential properties of the dried fruit by-products due to the presence of several phytochemicals with beneficial effects to human health [13].

A safety concern related to dried fruits is represented by health hazard due to natural/microbial toxins and by potential microbial spoilage during storage time. Dried fruits can be contaminated by kojic acid, several aflatoxins, ochratoxin-A, and occasionally other toxic compounds as zearalenone or patulin [14]. Moreover, high temperatures—used to dry fresh fruits—may generate the Maillard reaction to products due to potentially genotoxic browning and no-enzymatic reactions. For this reason, relatively low temperatures should be used to prevent fruit browning during appropriate fruit processing. Many studies are focused on innovative technologies in order to optimise the drying processes: modern freeze-drying technique has been shown to reduce enzymatic browning reactions (e.g., rate and extent) [15]. Moreover, sulphites (e.g., potassium metabisulphite and/or sulphur dioxide) are often used for their antioxidant and preservative capacity in dried fruit snacks. Due to their adverse effects to the asthmatic population [16], consumption of dried fruits with high levels of these substances could have negative effects for sensitive people [17,18].

The present research was aimed to study nutritional and phytochemical composition of freeze-dried fruits from four fruit species, two traditional commercial snacks (apple and goji) and two unconventional products (kaki and kiwifruit): nutritional traits (sugars), organic acids, total polyphenolic content (TPC), and the fingerprint of the main health-promoting phytochemicals by HPLC were studied. Furthermore, an *in vitro* antioxidant capacity screening was carried out. A Principal Component Analysis (PCA) was carried out as well as multivariate approach for setting a potential tool to analytically characterise these products. Finally, this research intended to stimulate large-scale commercial exploitation of dried fruit snacks as potential functional foods.

## 2. Materials and Methods

### 2.1. Plant Material and Dried Fruit Preparation

The investigated material consisted of dried fruits from four species: (i) apple, *Malus domestica* Borkh., two cultivars (cv), ‘Golden Delicious’ and ‘Camela’, identified as AG and AC, respectively; (ii) kiwifruit, *Actinidia deliciosa* (A.Chev.) C.F.Liang and A.R.Ferguson, cv ‘Hayword’, encoded as KH; (iii) kaki, *Diospyros kaki* L.F., cv ‘Fuyu’, called as CF; and (iv) goji, *Lycium barbarum* L., cv ‘Sweet’, identified as GS. Fruits were harvested during the 2018 season from a commercial orchard (Azienda Agricola Albertengo, Revello, Cuneo Province, Italy). After washing, fruits were stored in a 4 °C cold room until drying. Inedible parts were rejected.

Freeze-drying, a recent commercial drying technique, was used. In this technique, fruits are dried by water removal with dehydration (i.e., ice is sublimated in the plant materials by an air flow at temperature of 35–40 °C and humidity of 2% to 3%). The equipment (Model NWT- 25, North West Technology, Cuneo, Italy) was based on the “vaporization chain system” with a system power of 0.70–0.75 kW/h, a drying cycle capacity of 40 kg and a drying cycle time from 6 to 48 h.

### 2.2. Analytical Protocols and Methods

A more detailed description of the techniques and methods used for the extraction of bioactive compounds and HPLC analysis of dried fruits is reported in the Supplementary material (Tables S1 and S2).

#### 2.2.1. Spectrophotometric Analysis

The TPC (total polyphenol content) was measured by the Folin-Ciocalteu method [19,20]. The content of total phenolics was expressed as mg of gallic acid equivalents (GAE) per 100 g of dried weight (DW). Antioxidant capacity (AOC) was evaluated by the ferric reducing antioxidant power (FRAP) assay [21] and results were expressed as millimoles of ferrous iron (Fe^2+^) equivalents per kilogram of DW. The standard calibration curves were obtained using (i) gallic acid at concentration range of 0.02–0.1 mg/mL for TPC and (ii) FeSO_4_·7H_2_O at a concentration range of 100–1000 mmol/L for AOC.

An UV/Vis spectrophotometer (1600-PC, VWR International, Milan, Italy) single beam spectrophotometer was used to analyse TPC and antioxidant capacity (AOC).

#### 2.2.2. Chromatographic Analysis

Separation and identification of compounds were performed by HPLC analysis, using an Agilent 1200 HPLC - UV-Vis Diode Array Detector (Agilent Technologies, Santa Clara, CA, USA).

Chromatographic separation was performed on a Kinetex C18 column (4.6 × 150 mm, 5 μm, Phenomenex, Torrance, CA, USA), and a SphereClone NH2 column (4.6 × 250 mm, 5 μm, Phenomenex, Torrance, CA, USA). Different chromatographic conditions were utilsed to analyse dried fruit snacks according to the methods described and previously validated by Donno et al. [22] and Soifoini et al. [23], with some modifications.

Specific wavelengths were selected to identify and quantify more specific peaks and detection was performed by scanning from 190 to 600 nm.

Phytocomplex was intended as the sum of selected biomarkers identified comparing retention times and spectroscopic data with authentic standards (external standard calibration method) using the same chromatographic conditions following the “multi-marker approach” by Mok et al. [24]. The most important technique used for the characterisation of plant material is by measuring the concentration of one or very few markers or active components (“marker approach”). This approach is far from satisfactory, as the biological activity is due to more than one or two single chemical compounds. The “multi-marker approach” used in this research is the natural extension of the “marker approach”. In particular, it uses many, or even all, identified substances (chemical profile of the considered sample) to represent the whole sample. This approach is applied to many complex systems (e.g., herbs, herbal preparations, food supplements, foodstuffs). It is impossible to consider all the bioactive substances that may be included in the phytocomplex due to very high number of potential molecules; thanks to the multi-marker approach, the phytocomplex is approximated to the most important compounds with biological activity (demonstrated in literature). The higher the number of markers—the smaller the approximation will be. In this case, five phenolic classes were selected for the evaluation. Levels of vitamin C, monoterpenes, carotenoids, organic acids, and sugars were studied as well. Bioactive compound contents were expressed as mg/100 g of DW—except for sugars (expressed as g/100 g of DW) and carotenoids (expressed as µg/g of DW).

### 2.3. Statistical Analysis

Data were subjected to one-factor ANOVA test, and the mean values were compared with Tukey’s HSD post-hoc comparison test at *p* < 0.05 (*n* = 3). Data are expressed as mean value ± standard deviation (SD). Significant statistical differences (*p* < 0.05) are highlighted by different letters according to Tukey test.

Multivariate analysis (MVA) was performed on all of the samples. Fifteen objects (three repetitions for five samples) and 11 variables (content of nine chemical classes, TPC, antioxidant capacity) were included in the data matrix. Data were mean centred by Z-score scaling before MVA. Column-centred data were subjected to a Principal Component Analysis (PCA). All statistical calculations were performed with IBM SPSS Statistics 22.0 (IBM, Armonk, NY, USA).

## 3. Results and Discussion

### 3.1. Total Phenolics and Antioxidant Capacity

Few data are available on the antioxidant molecules and phenolic components in dried fruit snacks.

Folin–Ciocalteu reagent assay was used to determine TPC of analysed dried fruits. This method—despite the presence of many interferences—was used as a complementary technique in order to support and confirm chromatographic results, as reported in other studies [22,25]. In this study TPC values ranged from 210.90 ± 8.03 mg GAE/100 g DW (KH) to 872.58 ± 162.00 mg GAE/100 g DW (CF) as shown in Table 1. Values obtained in this study were similar to values reported by Vinson et al. [26] and Wu et al. [27]. In particular, dried apple snacks showed TPC of about 220 and 285 mg GAE/100g DW for ‘Golden Delicious’ and ‘Camela’ cultivars, respectively, similar to the TPC values (about 324 mg GAE/100g DW) reported by Alasalvar et al. [1].

Phenolics contribute to the fruit and vegetable antioxidant capacity showing a multitude of functional capacities, with many beneficial effects on human health [28]. Many methods can be used to determine antioxidant capacity (AOC) in plant material and derived-products [6]. In this research, FRAP assay was preliminarily utilised for the evaluation of antioxidant capacity in dried fruits as shown in other similar studies [29]. In this study, FRAP values ranged from 23.09 ± 0.74 mmol Fe^2+^/kg DW (GS) to 137.45 ± 12.60 mmol Fe^2+^/kg DW (CF) as shown in Table 1—in agreement with other studies [27,30]. In particular, apple and goji dried fruits showed AOC values similar to values reported by Donno et al. [29] and Pellegrini et al. [30], while no data on AOC kiwi and kaki dried fruits is available in scientific literature because they are innovative products not yet very widespread at commercial level—even if they present a high potential health-value.

This research is a preliminary study based on describing the composition of different dried fruits in order to assess their potential as health-promoting snacks. Comparing to data reported in other similar studies [26,31], values were much higher in dried fruits compared to their fresh counterparts due to concentration after drying or dehydration process. In any case, further analysis on fresh and dried fruits are necessary to confirm this preliminary hypothesis. The drying process causes a modification and/or loss of some specific compounds, but antioxidant capacity and TPC did not change during the process because these two parameters mainly depend on such large number of phenolic compounds (some of these molecules are yet to be identified) that no difference can be detected by multi-marker approach—even if the number of markers is high.

### 3.2. Phytochemical Composition

In this study, 37 compounds were selected as biomarkers for fingerprinting because of their nutritional and health-effective activity on humans [32]. This study added information on phytochemical profiles of the selected dried fruit snacks that could be considered as a potential natural source of bioactive molecules in the food industry.

**Figure 1:**
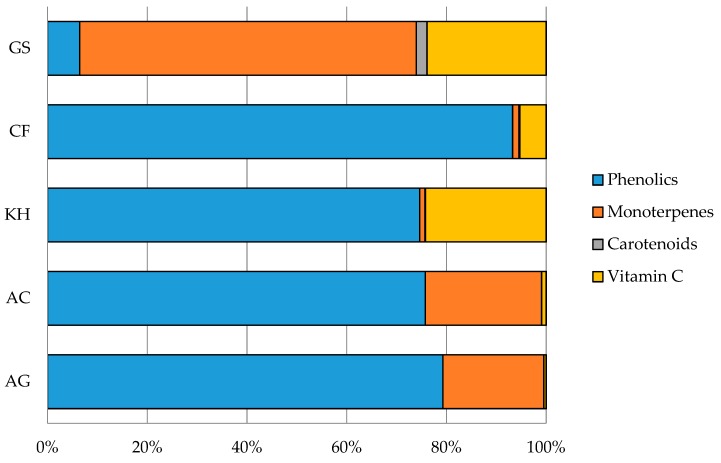
Phytocomplex of analysed dried fruit products. Mean values are given (*n* = 3). AG = *Malus domestica* Borkh., ‘Golden Delicious’; AC = *Malus domestica* Borkh., ‘Camela’; KH = *Actinidia deliciosa* (A.Chev.) C.F.Liang and A.R.Ferguson, ‘Hayword’; CF = *Diospyros kaki* L.F., ‘Fuyu’; GS = *Lycium barbarum* L., ‘Sweet’.

Chemical fingerprint of the analysed dried fruit snacks is reported from Table 2, Table 3, Table 4 and Table 5 (phenolics, monoterpenes, carotenoids and vitamin C, organic acids, and nutritional compounds as sugars, respectively) and in the Supplementary material (Table S3). In Figure 1, detected bioactive molecules were grouped into chemical classes for the evaluation of their contribution to the total phytocomplex. The most important class in apple, kiwi, and kaki dried fruits was phenolics (from 74.6% to 93.3%), expressed as the sum of catechins, flavonols, and phenolic acids, while monoterpenes were the first class in goji (GS) with a value of 67.5%. Kiwi and goji dried fruits showed a high vitamin C content (24.2% and 23.9%, respectively). Goji also presented an appreciable carotenoid content (about 2%).

**Figure 2:**
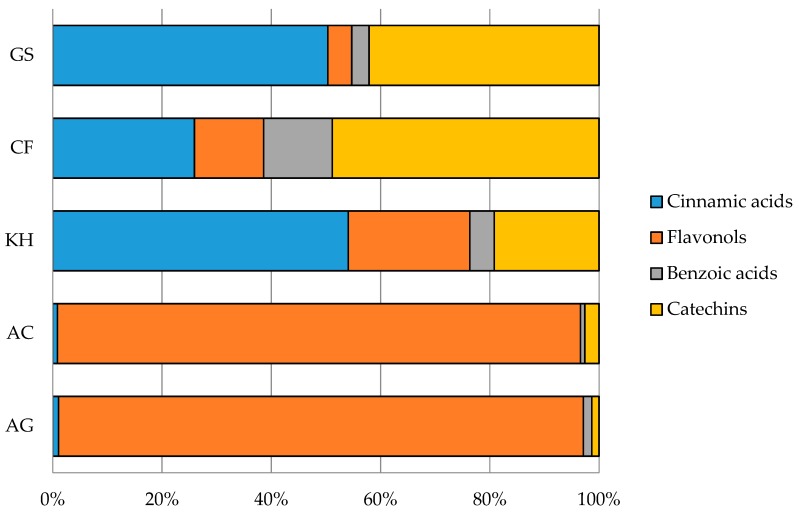
Phenolic composition of analysed dried fruit snacks. Mean values are shown (*n* = 3). AG = *Malus domestica* Borkh., ‘Golden Delicious’; AC = *Malus domestica* Borkh., ‘Camela’; KH = *Actinidia deliciosa* (A.Chev.) C.F.Liang and A.R.Ferguson, ‘Hayword’; CF = *Diospyros kaki* L.F., ‘Fuyu’; GS = *Lycium barbarum* L., ‘Sweet’.

Results identified the analysed dried fruit snacks as a very good source of phenolics in relation to other similar products [33,34,35,36]. Contribution of each phenolic class to total phenolic content is reported in Figure 2. Catechins were the most abundant class in kaki and goji snacks (48.9% and 42.1%, respectively), while phenolic acids (benzoic plus cinnamic acids) was the most important class in kiwi (58.6%) and goji (53.5%) dried fruits. Apple snacks showed a high flavonol content (more than 90% for both cultivars), but kiwi and kaki dried fruits presented a good content as well (22.3% and 12.7%, respectively).

No anthocyanins have been identified in analysed dried fruit snacks, because they are likely degraded to phenolic acids during the drying process as reported in other studies [37,38]. Indeed, structure of the fruit was changed by technological treatment (e.g., enzymatic and no-enzymatic reactions are modified in the tissues, as well as the conditions of the heat and mass exchange occuring in the plant material) [39]. However, some studies report that fruit drying during snack production does not significantly change the qualitative polyphenolic profile [40]; in particular, manufacturing process does not affect flavonoid levels [41].

In this research, each snack showed a specific polyphenolic composition characterised by the presence of one or more bioactive markers reported in Table 2.

Six phenolic acids—two of which being benzoic acid derivatives (gallic and ellagic acids) and four being derivatives from cinnamic acid (caffeic, chlorogenic, ferulic, and *p*-coumaric acids), along with five flavonols (hyperoside, isoquercitrin, quercetin, quercitrin, and rutin)—have been identified; (+)-catechin and (−)-epicatechin complete the polyphenolic composition of analysed dried fruits. Results showed that each dried fruit snack has a unique spectrum of phenolic compounds. The predominant phenolic compounds in apple snacks are phenolic acids (about 28 mg/100 g DW) and flavonols, in particular isoquercitrin (826.61 ± 58.39 mg/100 g DW), rutin (192.94 ± 34.58 mg/100 g DW), and quercitrin (118.75 ± 24.00 mg/100 g DW) as reported in other studies [42]. Cinnamic acids—especially chlorogenic and *p*-coumaric acids—are the main phenolics in kiwi and kaki (83.15 ± 2.76 mg/100 g DW and 80.33 ± 1.42 mg/100 g DW, respectively) together with catechins, in particular (−)-epicatechin (20.93 ± 0.48 mg/100 g DW for kiwi and 150.68 ± 0.73 mg/100 g DW for kaki). Caffeic, ferulic and *p*-coumaric acids could be involved in the same degradation and modification processes during technological steps, but the use of freeze-drying reduces this possibility as demonstrated by Donno et al. [29]. Moreover, quercitrin is the predominant flavonol in dried kiwi and kaki snacks (29.24 ± 0.64 mg/100 g DW and 30.29 ± 0.40 mg/100 g DW, respectively) as shown by Chin et al. [43]. Flavonols and chlorogenic acid may be considered the main phenolics responsible for *in vitro* anti-cancer activity (e.g., against liver, colon, breast, and lung cancer), as recently reported by Li et al. [44]. All six phenolic acids, selected as health-promoting markers, have been identified in goji dried fruits—in particular ferulic acid (9.66 ± 0.46 mg/100 g DW). Moreover, good levels of catechins have been detected (11.90 ± 0.96 mg/100 g DW), while flavonols were observed in trace (<2 mg/100 g DW) with levels similar to other researches [29,45].

Monoterpenes represent an important bioactive class in the analysed dried snack phytocomplex, in particular limonene in dried apples (311.48 ± 31.30 mg/100 g DW), and phellandrene (185.12 ± 8.22 mg/100 g DW) and γ-terpinene (57.87 ± 3.41 mg/100 g DW) in dried goji products as shown in Table 3. Monoterpenes—molecules with antitumoral and antibacterial capacity found in the essential plant oils—were observed in trace in kiwi and kaki snacks (from 2 to 5 mg/100 g DW). Plant terpenoids are non-nutritive dietary substances, often used for their anti-inflammatory and antioxidant capacity together with high aromatic qualities [46]. Recent studies reported dried fruit chemopreventive actions against different types of cancers [47] due to these molecules.

Six carotenoids (α-carotene, β-carotene, and β-cryptoxanthin which act as provitamin A; lutein, lycopene, and zeaxanthin, which does not have provitamin A activity), have been observed in the considered dried fruits (Table 4). β-carotene was the most abundant in dried goji fruits (35.30 ± 0.31 μg/g DW), followed by kaki (10.26 ± 0.33 μg/g DW), and kiwi (9.39 ± 0.85 μg/g DW) snacks. Dried goji products showed high contents of lutein (46.49 ± 0.62 μg/g DW) and zeaxanthin (793.64 ± 1.84 μg/g DW) as well, confirming the results reported by Zhong et al. [45]. α-carotene (from 0.75 to 13 μg/g DW) and lycopene (from 0.22 to 0.76 μg/g DW) were detected only in dried kiwi and goji. Dried kaki fruits were rich in β-cryptoxanthin (26.36 ± 0.81 μg/g DW), while these compounds have been detected in dried apples as trace amounts (< 10 μg/g DW) as shown in other studies [34,48]. The drying process may cause a lowering of carotenoid levels in dried fruit snacks since carotenoids are thermolabile [15]. In this study, carotenoid concentration (e.g., levels of lutein, β-carotene and β-cryptoxanthin) were significantly increased by drying compared to their fresh counterparts because of the phytochemical concentration due to the removal of water [6].

Vitamin C values were reported in Table 4 as the sum of dehydroascorbic and ascorbic acids [49]. In this research goji and kiwi snacks presented a high vitamin C content (104.58 ± 3.65 mg/100 g DW and 49.85 ± 0.49 mg/100 g DW, respectively) as shown by Donno et al. [29] and Kaya et al. [40]. Other analysed snacks showed high vitamin C values (about 8–18 mg/100 g DW) as reported by previous similar studies [34,50].

Processing and storage influence organic acid levels in fruits—even if they show a high stability when compared to flavour compounds and pigments [30]. Moreover, they are antioxidant compounds as well, sometimes used in pharmacological trials, as shown by Eyduran et al. [51]. Table 5 showed that dried apple snacks contain high levels of organic acids, as tartaric acid (from 420 to 490 mg/100 g DW) and malic acid (from 50 to 140 mg/100 g DW); these compounds, together with fibre, may synergistically exert a biological action in order to maintain digestive system in good health conditions. They may increase the bioavailability of mineral elements, such as iron and calcium, in the diet [52]. Dried kiwi fruits, as well as kaki and goji, contain high contents of quinic acid (881.21 ± 0.94 mg/100 g), that can be metabolised to hippuric acid, which is useful to alleviate infections in the urinary tracts [36].

The sugar pattern is reported in Table 5. Dried apple fruits showed the highest sugar content (from 71 to 83 g/100 g DW), expressed as sum of fructose (36.17 ± 3.13 g/100 g DW), glucose (35.44 ± 3.47 g/100 g DW), and sucrose (5.65 ± 2.88 g/100 g DW) according to previous researches [33,42]. Dried goji snacks showed a sugar level of about 45 g/100 g DW as shown in previous researches [45,50], while dried kaki and kiwi snacks showed small amounts of sugars (15.26 ± 1.26 g/100 g DW and 8.67 ± 1.27 g/100 g DW, respectively) with a clear positive effect on carbohydrate metabolism if compared to traditional dried snacks.

### 3.3. Biomarker PCA Evaluation

**Figure 3:**
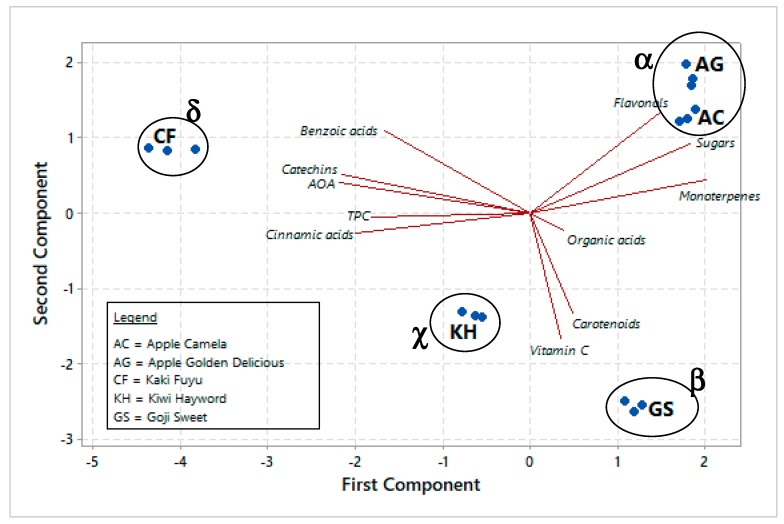
PCA biplot of dried fruit products (three replications for each sample). The ellipses only define the category position in the PCA biplot with no statistical meaning. Biplot shows correlation among nutri—nutraceutical properties and PCs.

Single markers belonging to the same chemical class were grouped for multivariate analysis. PCA reduced the initial eleven variables to three PCs (98.4% of total system variance) and it placed the five dried fruit snacks in the PCA biplot (Figure 3) related to nutraceutical and nutritional properties. The system information was well represented by PC1 and PC2 (76.9% of total variance); four category groups were identified by PCA (Figure 3), following the fingerprinting data; the groups were named α (apple), β (goji), χ (kiwi), and δ (kaki).

PCA biplot showed a correlation between some phenolic classes (phenolic acids, catechins, and flavonols), TPC, AOC, monoterpenes, sugars and PC1 (49.4% of total variance), and a correlation between vitamin C, organic acids, carotenoids and PC2 (27.5% of total variance) as well. Phenolics (in particular, phenolic acids and catechins), carotenoids, and vitamin C—as well as organic acids and nutritional compounds as sugars—showed a high power for discriminating different samples according to other researches [33,50].

PCA classification characterised the analysed products and showed important information on biomarkers with important effects on the phytocomplex composition. Different markers could be applied to the dried fruit snack composition control; in particular, PCA biplot showed that products included in the α group (dried apple snacks) present the highest amount of flavonols and monoterpenes together with the highest amount of sugars. Kaki products are mainly characterised by phenolics and antioxidant capacity, while kiwi and goji dried fruits were essentially characterized by high levels of vitamin C. Chromatographic fingerprinting coupled to chemometrics could be a potential effective tool for evaluation, differentiation and quality control of dried fruit products, in order to obtain label certifications for their valorisation; however, analysis of other commercial samples with other specific traits (e.g., different nature and origin) are necessary to fully demonstrate that the proposed approach may be applicable. Little data is available on the ADME (absorption, distribution, metabolism, and excretion) of these biologically active molecules in human body (e.g., polyphenol bioaccessibility and bioavailability); however, *in vitro* model information should be correlated to human studies and animal models. For this reason, the identification of the metabolites of these bio-compounds after urinary excretion may be very important together with the application of cellular models [53]. These data may demonstrate their supposed biological relevance in nutrition and human health.

## 4. Conclusions

This research has been carried out to determine the phytochemical profiles in relation to their antioxidant and/or health-promoting activity in the analysed dried fruits. In this study results showed that unconventional snacks—based on kiwi and kaki—are characterised by high levels of phenolic acids, flavonoids, and carotenoids. In this research dried fruits have proved to be nutritional snacks comparable to fresh fruits—with a smaller serving size—and they could be inserted in the fresh fruit recommendations. This study may help to increase interest in scientific validation and development of potential functional foods from dried fruits—products already commercially widespread—even if not yet completely characterized in their chemical composition and potential biological activity. However, more sophisticated chemical studies (e.g., characterisation by mass spectrometry) and clinical trials are requested to confirm and validate their health benefits in order to obtain high quality and safety for consumers.

## Figures and Tables

**Table 1:** Total phenolics (TPC) and antioxidant capacity (AOC) of analysed dried fruits.

Sample	Cultivar	ID	TPC	AOC
			(mg GAE/100 g DW)	(mmol Fe^2+^/kg DW)
*Malus domestica*	Golden Delicious	AG	220.47 ± 20.56 ^a^	24.01 ± 1.52 ^a^
*Malus domestica*	Camela	AC	285.89 ± 29.64 ^a^	26.28 ± 0.35 ^a^
*Actinidia deliciosa*	Hayword	KH	210.90 ± 8.03 ^a^	32.66 ± 4.39 ^a^
*Diospyros kaki*	Fuyu	CF	872.58 ± 162.00 ^c^	137.45 ± 12.60 ^b^
*Lycium barbarum*	Sweet	GS	502.36 ± 71.22 ^b^	23.09 ± 0.74 ^a^

Data (*n* = 3) are expressed as mean value and standard deviation (SD). Significant statistical differences (*p* < 0.05) are highlighted by different letters (a–c). GAE—gallic acid equivalent; DW—dried weight.

**Table 2:** Phenolic compound fingerprint of considered dried fruits.

**ID**	**Cinnamic Acids**					
	**Caffeic Acid**	**Chlorogenic Acid**	***p*** **-Coumaric Acid**	**Ferulic Acid**		
	**(mg/100 g DW)**					
AG	n.d.	4.80 ± 0.12 ^b^	n.d.	9.83 ± 0.70 ^c^		
AC	n.d.	4.33 ± 0.12 ^b^	n.d.	6.28 ± 0.21 ^b^		
KH	4.03 ± 0.45 ^c^	53.40 ± 0.75 ^d^	22.35 ± 0.78 ^b^	3.36 ± 0.83 ^a^		
CF	2.28 ± 0.65 ^b^	52.08 ± 0.26 ^c^	22.08 ± 0.29 ^b^	3.89 ± 0.23 ^a^		
GS	1.14 ± 0.02 ^a^	1.02 ± 0.26 ^a^	2.42 ± 0.62 ^a^	9.66 ± 0.46 ^c^		
**ID**	**Flavonols**					
	**Hyperoside** **(quercetin-3-galactoside)**	**Isoquercitrin** **(quercetin-3-β-d-glucoside)**	**Quercetin**	**Quercitrin** **(quercetin-3-rhamnoside)**	**Rutin** **(quercetin-3-*O*-rutinoside)**	
	**(mg/100 g DW)**					
AG	91.09 ± 4.37 ^b^	848.81 ± 4.54 ^b^	5.88 ± 0.50 ^c^	140.65 ± 0.47 ^c^	223.90 ± 3.29 ^c^	
AC	105.35 ± 3.47 ^c^	804.42 ± 83.82 ^b^	1.52 ± 0.49 ^b^	96.86 ± 1.31 ^b^	161.99 ± 10.17 ^b^	
KH	n.d.	n.d.	1.05 ± 0.51 ^ab^	29.24 ± 0.64 ^a^	3.91 ± 0.44 ^a^	
CF	1.43 ± 0.68 ^a^	1.99 ± 0.61 ^a^	2.21 ± 0.46 ^b^	30.29 ± 0.40 ^a^	3.39 ± 0.66 ^a^	
GS	0.82 ± 0.03 ^a^	n.d.	0.24 ± 0.04 ^a^	n.d.	0.17 ± 0.01 ^a^	
**ID**	**Benzoic Acids**		**Catechins**			
	**Ellagic acid**	**Gallic acid**	**Catechin**	**Epicatechin**		
	**(mg/100 g DW)**		**(mg/100 g DW)**			
AG	10.15 ± 0.12 ^d^	10.82 ± 3.46 ^b^	10.44 ± 0.55 ^b^	7.71 ± 0.11 ^a^		
AC	8.55 ± 0.75 ^c^	0.99 ± 0.03 ^a^	21.63 ± 1.94 ^c^	10.36 ± 0.35 ^b^		
KH	4.31 ± 0.62 ^b^	2.54 ± 0.33 ^a^	8.57 ± 0.57 ^b^	20.93 ± 0.48 ^c^		
CF	15.71 ± 0.59 ^e^	23.26 ± 0.52 ^c^	0.75 ± 0.54 ^a^	150.68 ± 0.73 ^d^		
GS	0.14 ± 0.02 ^a^	0.75 ± 0.09 ^a^	3.14 ± 0.13 ^a^	8.76 ± 0.85 ^a^		

Data (*n* = 3) are shown as mean value ± standard deviation (SD). Significant statistical differences (*p* < 0.05) are highlighted by different letters (a–e). Results are expressed as mg/100 g DW. AG—*Malus domestica* Borkh., ‘Golden Delicious’; AC—*Malus domestica* Borkh., ‘Camela’; KH—*Actinidia deliciosa* (A.Chev.) C.F.Liang and A.R.Ferguson, ‘Hayword’; CF—*Diospyros kaki* L.F., ‘Fuyu’; GS—*Lycium barbarum* L., ‘Sweet’; DW—dried weight; n.d.—not detected.

**Table 3:** Monoterpenes in analysed dried fruits.

ID	Monoterpenes				
	Limonene	Phellandrene	Sabinene	γ-Terpinene	Terpinolene
	(mg/100 g DW)				
AG	299.51 ± 36.62 ^b^	2.86 ± 0.59 ^a^	43.48 ± 6.58 ^ab^	0.60 ± 0.31 ^ab^	1.59 ± 0.00 ^a^
AC	323.45 ± 26.05 ^b^	3.70 ± 0.68 ^a^	41.36 ± 5.15 ^a^	5.97 ± 2.96 ^b^	1.57 ± 0.01 ^a^
KH	1.68 ± 0.32 ^a^	n.d.	n.d.	0.45 ± 0.25 ^a^	n.d.
CF	1.83 ± 0.40 ^a^	n.d.	n.d.	0.56 ± 0.34 ^ab^	1.82 ± 0.15 ^b^
GS	n.d.	185.12 ± 8.22 ^b^	52.13 ± 1.52 ^b^	57.87 ± 3.41 ^c^	n.d.

Data (*n* = 3) are shown as mean value ± standard deviation (SD). Significant statistical differences (*p* < 0.05) are highlighted by different letters (a–c). Results are expressed as mg/100 g DW. AG—*Malus domestica* Borkh., ‘Golden Delicious’; AC—*Malus domestica* Borkh., ‘Camela’; KH—*Actinidia deliciosa* (A.Chev.) C.F.Liang and A.R.Ferguson, ‘Hayword’; CF—*Diospyros kaki* L.F., ‘Fuyu’; GS—*Lycium barbarum* L., ‘Sweet’; DW—dried weight; n.d.—not detected.

**Table 4:** Carotenoids and vitamin C in analysed dried fruits.

ID	Carotenoids						Vitamin C	
	α-Carotene	β-Carotene	β-Cryptoxanthin	Lutein	Lycopene	Zeaxanthin	Ascorbic Acid	Dehydroascorbic Acid
	(µg/g DW)						(mg/100 g DW)	
AG	n.d.	4.14 ± 0.49 ^a^	n.d.	2.33 ± 0.45 ^a^	n.d.	2.62 ± 0.34 ^b^	1.97 ± 0.04 ^a^	6.36 ± 2.94 ^a^
AC	n.d.	5.16 ± 0.81 ^a^	n.d.	2.05 ± 0.32 ^a^	n.d.	2.58 ± 0.56 ^b^	1.99 ± 0.09 ^a^	13.00 ± 1.72 ^b^
KH	12.88 ± 0.58 ^b^	9.39 ± 0.85 ^b^	n.d.	1.98 ± 0.15 ^a^	0.22 ± 0.02 ^a^	0.17 ± 0.01 ^a^	42.81 ± 0.33 ^d^	7.04 ± 0.16 ^a^
CF	n.d.	10.26 ± 0.33 ^b^	26.36 ± 0.81 ^a^	15.02 ± 0.79 ^b^	n.d.	11.47 ± 0.16 ^c^	13.29 ± 0.49 ^b^	4.29 ± 0.49 ^a^
GS	0.75 ± 0.10 ^a^	35.30 ± 0.31 ^c^	60.63 ± 1.17 ^b^	46.49 ± 0.62 ^c^	0.76 ± 0.15 ^b^	793.64 ± 1.84 ^d^	37.94 ± 2.37 ^c^	66.64 ± 1.28 ^c^

Data (*n* = 3) are shown as mean value ± standard deviation (SD). Significant statistical differences (*p* < 0.05) are highlighted by different letters (a–d). Results are expressed as μg/g DW for carotenoids and mg/100 g DW for vitamin C. AG—*Malus domestica* Borkh., ‘Golden Delicious’; AC—*Malus domestica* Borkh., ‘Camela’; KH—*Actinidia deliciosa* (A.Chev.) C.F.Liang and A.R.Ferguson, ‘Hayword’; CF—*Diospyros kaki* L.F., ‘Fuyu’; GS—*Lycium barbarum* L., ‘Sweet’; DW—dried weight; n.d.—not detected.

**Table 5:** Organic acids and sugars in analysed dried fruit snacks.

ID	Organic Acids						Sugars		
	Citric Acid	Malic Acid	Oxalic Acid	Quinic Acid	Succinic Acid	Tartaric Acid	Fructose	Glucose	Sucrose
	(mg/100 g DW)						(g/100 g DW)		
AG	n.d.	139.26 ± 14.69 ^b^	8.25 ± 3.60 ^ab^	192.01 ± 63.85 ^bc^	n.d.	423.58 ± 174.60 ^b^	37.40 ± 3.18 ^c^	37.95 ± 3.05 ^d^	7.89 ± 1.96 ^b^
AC	n.d.	49.86 ± 54.47 ^a^	13.19 ± 2.11 ^bc^	261.84 ± 56.42 ^c^	n.d.	489.26 ± 75.94 ^b^	34.94 ± 3.15 ^c^	32.92 ± 1.32 ^c^	3.42 ± 1.35 ^a^
KH	283.67 ± 0.52 ^c^	223.16 ± 0.50 ^c^	43.96 ± 0.56 ^d^	881.21 ± 0.94 ^d^	431.80 ± 0.50 ^c^	n.d.	3.42 ± 0.54 ^a^	3.85 ± 0.39 ^a^	1.40 ± 0.34 ^a^
CF	60.34 ± 0.35 ^a^	24.16 ± 0.56 ^a^	3.96 ± 0.48 ^a^	90.61 ± 0.28 ^ab^	17.19 ± 0.45 ^a^	13.87 ± 0.22 ^a^	5.43 ± 0.45 ^a^	7.40 ± 0.25 ^a^	2.44 ± 0.56 ^a^
GS	67.04 ± 0.51 ^b^	28.90 ± 0.30 ^a^	17.35 ± 0.09 ^c^	62.16 ± 0.76 ^a^	67.65 ± 0.66 ^b^	51.71 ± 0.57 ^a^	20.83 ± 0.90 ^b^	20.39 ± 0.51 ^b^	2.77 ± 1.26 ^a^

Data (*n* = 3) are presented as mean value ± standard deviation (SD). Significant statistical differences (*p* < 0.05) are highlighted by different letters (a–d). Results are expressed as mg/100 g DW for organic acids and g/100 g DW for sugars. AG—*Malus domestica* Borkh., ‘Golden Delicious’; AC—*Malus domestica* Borkh., ‘Camela’; KH—*Actinidia deliciosa* (A.Chev.) C.F.Liang and A.R.Ferguson, ‘Hayword’; CF—*Diospyros kaki* L.F., ‘Fuyu’; GS—*Lycium barbarum* L., ‘Sweet’; DW—dried weight; n.d.—not detected.

## Supplementary Materials

The following are available online at https://www.mdpi.com/2076-3921/8/9/396/s1, Table S1: Protocols for the extraction of bioactive compounds and nutritional substances, Table S2: Chromatographic conditions of the used HPLC methods, Table S3: Phytocomplex of considered dried fruits.
