# Supplementary material for: Traditional and Unconventional Dried Fruit Snacks as a Source of Health-Promoting Compounds
Source: Antioxidants (Basel). 2019 Sep 13;8(9):396. doi: 10.3390/antiox8090396 (PMC6770932; doi:10.3390/antiox8090396)
Supplement: Supplementary file 1 [file antioxidants-08-00396-s001.pdf]

antioxidants

Table S1. Protocols for the extraction of bioactive compounds and nutritional substances.

Chemical class	Sample Weight	Extraction Solvents	Time of Extraction	Homogenisation	Centrifugation	Number of Successive Extraction	Other Actions
Polyphenolic compounds	10 g	methanol : bi-distilled water, 95:5 v/v, pH adjusted with 1.5 mL of 37% HCl (25 mL)	60 min in the dark	1 min	15 min at 3,000 rpm	3	/
Organic acids, sugars, and monoterpenes	5 g	95% ethanol (25 mL)	30 min in the dark	1 min	10 min at 4,000 rpm	2	/
Vitamin C	5 g	0.1 M citric acid, 2 mM EDTA disodium salt, and 4 mM sodium fluoride in methanol – water, 5:95 v/v (10 mL)	20 min in the dark	1 min	10 min at 4,000 rpm	2	Supernatant acidified with 4 N HCl (pH = 2.2–2.4) and centrifuged for 5 min at 12,000 rpm at 4 °C
Carotenoids	5 g	methanol : tert-butyl methyl ether, 70:30 v/v (35 mL)	30 min in the dark	1 min	15 min at 4,000 rpm	2	/

Table S2. Chromatographic conditions of the used HPLC methods.

Method	Classes of Interest	Stationary Phase	Mobile Phase	Wavelength *(nm)*
A	cinnamic acids, flavonols	KINETEX – C18 column (4.6 × 150 mm, 5 μm)	A: 10 mM KH_2PO_4/H_3PO_4, pH = 2.8<br>B: CH_3CN	330
B	benzoic acids, catechins	KINETEX – C18 column (4.6 × 150 mm, 5 μm)	A: $H_2O/CH_3OH/HCOOH$ (5:95:0.1 v/v/v), pH = 2.5<br>B: $CH_3OH/HCOOH$ (100:0.1 v/v)	280
C	monoterpenes	KINETEX – C18 column (4.6 × 150 mm, 5 μm)	A: H_2O<br>B: CH_3CN	250
D	organic acids	KINETEX – C18 column (4.6 × 150 mm, 5 μm)	A: 10 mM KH_2PO_4/H_3PO_4, pH = 2.8<br>B: CH_3CN	214
E	vitamins	KINETEX – C18 column (4.6 × 150 mm, 5 μm)	A: 5 mM $C_{16}H_{33}N(CH_3)_3Br$/50 mM KH_2PO_4, pH = 2.5<br>B: CH_3OH	261, 348
F	sugars	SphereClone – NH_2 column (4.6 × 250 mm, 5 μm)	A: H_2O<br>B: CH_3CN	267, 286
G	carotenoids	KINETEX – C18 column (4.6 × 150 mm, 5 μm)	A: ACN<br>B: CH_3OH<br>C: CH_2Cl_2	450

Elution conditions

Method A, gradient analysis: 5%B to 21%B in 17 min + 21%B in 3 min (2 min conditioning time); flow: 1.5 mL min^{-1}

Method B, gradient analysis: 3%B to 85%B in 22 min + 85%B in 1 min (2 min conditioning time); flow: 0.6 mL min^{-1}

Method C, gradient analysis: 30%B to 56%B in 15 min + 56%B in 2 min (3 min conditioning); flow: 1.0 mL min^{-1}

Method D, gradient analysis: 5%B to 14%B in 10 min + 14%B in 3 min (2 min conditioning time); flow: 0.6 mL min^{-1}

Method E, isocratic analysis: ratio of phase A and B: 95:5 in 10 min (5 min conditioning time); flow: 0.9 mL min^{-1}

Method F, isocratic analysis: ratio of phase A and B: 5:85 in 12 min (3 min conditioning time); flow: 0.5 mL min^{-1}

Method G, isocratic analysis: ratio of phase A, B and C: 75:20:5 in 20 min (5 min conditioning time); flow: 1.0 mL min^{-1}

Table S3. Phytocomplex of considered dried fruits.

ID	Polyphenols (mg/100 g DW)	Monoterpenes (mg/100 g DW)	Carotenoids (µg/g DW)	Vitamin C (mg/100 g DW)	Organic Acids (mg/100 g DW)	Sugars (g/100 g DW)
AG	1364.07 ± 18.24[d]	348.03 ± 44.10[c]	9.10 ± 1.29[a]	8.33 ± 2.98[a]	763.11 ± 256.73[c]	83.24 ± 8.19[d]
AC	1222.28 ± 102.67[d]	376.04 ± 34.85[c]	9.80 ± 1.69[a]	14.98 ± 1.81[ab]	814.16 ± 188.95[c]	71.28 ± 5.82[cd]
KH	153.71 ± 6.40[b]	2.12 ± 0.57[a]	24.64 ± 1.62[b]	49.85 ± 0.49[c]	1863.80 ± 3.01[d]	8.67 ± 1.27[a]
CF	310.03 ± 6.63[c]	4.21 ± 0.89[a]	63.11 ± 2.08[c]	17.58 ± 0.98[b]	210.13 ± 2.33[a]	15.26 ± 1.26[b]
GS	28.27 ± 2.53[a]	295.13 ± 13.15[b]	937.58 ± 4.18[d]	104.58 ± 3.65[d]	294.82 ± 2.89[b]	43.99 ± 2.67[c]

Data (n = 3) are shown as mean value ± standard deviation (SD). Significant statistical differences ($p < 0.05$) are highlighted by different letters (a-d). AG = *Malus domestica* Borkh., 'Golden Delicious'; AC = *Malus domestica* Borkh., 'Camela'; KH = *Actinidia deliciosa* (A.Chev.) C.F.Liang and A.R.Ferguson, 'Hayword'; CF = *Diospyros kaki* L.F., 'Fuyu'; GS = *Lycium barbarum* L., 'Sweet'. DW = dried weight.
